# Assessment of knowledge, attitudes, and practices regarding sun exposure and sun protection among female students living in high-altitude areas, Abha, Saudi Arabia: a cross-sectional study

**DOI:** 10.7717/peerj.20576

**Published:** 2026-01-07

**Authors:** Maha Ali, Bayapa Reddy Narapureddy

**Affiliations:** Department of Public Health, College of Applied Medical Sciences, King Khalid University, Abha, Assir, Saudi Arabia

**Keywords:** Sun exposure, Sun protection factor, Skin neoplasms, Solar radiation exposure, High altitude, Ultraviolet rays

## Abstract

**Background:**

Ultraviolet (UV) rays are more intense at high-altitude regions. Exposure to intense UV rays is a major risk factor for skin damage and skin cancer. The university students (young adults) have limited awareness of sun protection measures, leading to persistent gaps in their knowledge, attitudes, and practices (KAP). This study evaluates the KAP of sun exposure and protection measures among female students at King Khalid University in Abha, Saudi Arabia.

**Objectives:**

To assess students’ understanding of sun exposure risks and protective measures, to identify students’ attitudes toward sun safety, and to determine students’ sun exposure and protection practices.

**Methods:**

A descriptive cross-sectional study was conducted on 328 female students from various academic programs using a stratified random sampling method. Data were collected using a validated questionnaire assessing sociodemographic details, knowledge, attitudes, and practices toward sun exposure and sun protective measures at high altitudes. Statistical analysis was conducted using SPSS V.27, with descriptive statistics determining frequency and percentages, and inferential statistics assessing relationships between KAP variables.

**Results:**

The majority of participants (77.1%) were aware of the stronger sun rays at high altitudes, and 72.9% recognized high UV intensity. However, misconceptions persisted, with 55.2% believing sunscreen use before age 30 causes skin darkening. While 66.5% reported using sun protection, inconsistencies in sunscreen use and other protective behaviors were observed. Statistically significant relationships were found between academic year and sun-related knowledge (*p* < 0.001), as well as between knowledge, attitudes, and practices.

**Conclusion:**

Despite good awareness and positive attitudes toward sun protection, practical application remains inconsistent. Targeted educational campaigns and culturally appropriate interventions are essential to bridging the gap between knowledge and practice. Future studies should focus on evaluating intervention effectiveness and long-term behavioral changes.

## Introduction

Skin cancer is one of the most commonly diagnosed cancers across the globe. Skin cancer ranks as the 17th most prevalent cancer globally and is also the 14th most common cancer among both men and women worldwide (Wang, Gao & Zhang, 2025). The current epidemiological data show that its occurrence is steadily rising worldwide. In the year 2022, Global healthcare reports indicated that 1.5 million newly diagnosed skin cancer cases, 331,722 belonging to malignant melanoma (Wang, Gao & Zhang, 2025). Exposure to ultraviolet (UV) radiation remains the main risk factor for skin cancer (Armstrong & Kricker, 2001). Chronic exposure to ultraviolet radiation results in premature aging, skin pigmentary changes like melasma and lentigines, and causes different skin cancers (Rigel, Russak & Friedman, 2010; Krutmann et al., 2017). The World Health Organization (WHO) states that several negative health effects result from UV radiation exposure, including sunburn, along with skin aging, which also suppresses immunity and damages eyesight by causing cataract (Watson, Holman & Maguire-Eisen, 2016).

In Saudi Arabia, the Ministry of Health has highlighted six major health risks related to excessive sun exposure, which include skin cancer, cataracts, sunstroke, skin burns with premature wrinkles, and deterioration of the immune system (Ministry of Health, Kingdom of Saudi Arabia, 2025). Saudi society demonstrates high awareness about sun exposure risks, yet fails to maintain adequate sun protection measures. Different attitudes toward sun protection products combined with cultural influences on sun-related behaviors explain this discrepancy (Holman et al., 2018; Glanz & Mayer, 2005).

Skin cancer development involves genetic susceptibility, but chronic excessive UV exposure stands out as a major determinant. When skin cells are exposed to UV radiation, they experience DNA damage that can eventually lead to mutations, which trigger cancerous transformations (Narayanan, Saladi & Fox, 2010). The immune system becomes weaker due to UV radiation, which leads to decreased ability of the body to identify and destroy abnormal cells (Narayanan, Saladi & Fox, 2010). Skin sagging, roughness, and hyperpigmented spots develop from prolonged UV exposure because this exposure leads to collagen and elastin fiber breakdown (Fisher et al., 2002). The American Cancer Society (ACS) has found that in the United States, the estimated lifetime risk of developing cancer is approximately 40% for men and 39% for women, meaning about 40 out of every 100 men and 39 out of every 100 women will be diagnosed with cancer during their lifetime (American Cancer Society, 2025). According to local statistics, skin cancer ranks as the ninth most frequently occurring cancer type in Saudi Arabia (Saudi Health Council & National Cancer Center, 2023). UV-related skin damage remains preventable, yet protective behaviors like sunscreen application and sun exposure reduction during peak hours remain uncommon (Alshammrie et al., 2022).

Solar radiation exposure increases the threat of UV-related skin damage and skin cancer in high-altitude environments. As elevation increases by 1,000 m (3,280 ft), UV intensity increases by about 10–12% which substantially increases the danger of sun exposure  (Dana-Farber, 2015). At higher altitudes, the thinner atmosphere allows more harmful UV rays to penetrate the skin (McKenzie et al., 2007). In snowy or icy regions, UV rays bounce off surfaces, which leads to exposure levels doubling and raises sunburn risk, along with long-term skin damage risks (Koumaki et al., 2024). Research indicates that people residing in or regularly visiting high-altitude areas face an increased risk of skin cancer development because of extended UV exposure of greater intensity (Andersen et al., 2010). People living in high-altitude areas need to implement rigorous sun protection strategies by using SPF 50+ sunscreen and wearing protective clothing and UV-blocking sunglasses while also limiting exposure to sunlight during peak hours.

## Rationale for the Study

Young adult females represent a key population for investigating sun exposure patterns. Establishing sun protection practices during this developmental stage is crucial for preventing future skin damage and reducing the risk of skin cancer. As prospective healthcare providers, these students play an essential role in promoting sun safety awareness and delivering patient education. Therefore, assessing their knowledge, attitudes, and practices (KAP) regarding sun exposure and sun protection measures is vital for developing targeted educational interventions. However, persistent challenges include widespread misconceptions, inadequate awareness of sunscreen use, and insufficient emphasis on year-round sun safety practices (Friedli et al., 2024).

This study contributes novel evidence from a high-altitude region (Abha, >2,200 m above sea level), where environmental factors interact with unique cultural and behavioral determinants. The combination of traditional Saudi attire, limited outdoor activity due to extreme temperatures, and heightened UV intensity creates a distinctive context for examining sun protection behaviors. Although several international studies have explored KAP related to sun exposure and protective measures, there remains a significant research gap in Saudi Arabia, particularly regarding studies that examine the convergence of environmental, cultural, and behavioral factors among female university students (Flemban et al., 2025; Chavez et al., 2025).

This study aims to address this gap by evaluating the KAP of female students at King Khalid University (KKU), identifying knowledge deficits, and proposing evidence-based health promotion interventions to enhance sun protection behaviors in this vulnerable population.

Objectives: The study evaluated female medical students’ knowledge about sun exposure risks and sun protection strategies. This research will examine female students’ attitudes toward sun safety measures. This study aims to identify sun exposure patterns and protective behaviors among female students at King Khalid University.

### Methods and Materials

A descriptive cross-sectional study to examine female students’ knowledge and attitudes as well as their practices about sun exposure and sun protection methods in high-altitude areas like Abha, Saudi Arabia. This study was conducted from April 2025 to July 2025.

### Sampling and sample size

The research utilized a stratified proportional random sampling method to select female students from three academic programs (Public Health, Nursing, and Sciences and Literature) obtained from the college registration department. The required minimum sample size was determined by using Raosoft’s online sample size calculator (Raosoft Inc., 2024) based on a 95% confidence level and a 5% margin of error for a population of 2,236 female students with an expected response rate of 50%, resulting in a sample size of 328. Participants were drawn in proportion to their representation in the university population to ensure adequate representation across programs and academic years.

Inclusion: Female students enrolled in KKU programs (Sciences and Literature, Nursing, Public Health) during the study period and able to complete a self-administered questionnaire approved by the KKU Ethics Committee.

Exclusion: Individuals not in the defined programs, male students, and those not consenting or not completing the questionnaire adequately; no imputation was performed. Pilot respondents (*n* = 33) were excluded from the final analysis.

Methods: Official student lists by program obtained from the college registration department at KKU formed the sampling frame for Sciences and Literature, Nursing, and Public Health female students (total 2,236). Stratified proportional random sampling with proportional allocation from each program was applied to the registration lists to draw 328 participants; no imputation was performed, with proportional sampling across programs to avoid over- or under-representation of strata, ensuring representation across academic years and programs and reducing selection bias.

### Data collection and tools

Recruitment and consent: Eligible students received a secure survey link *via* university email and WhatsApp groups. After reading the study information, participants provided e-consent to access the questionnaire. The system prevented duplicate responses *via* single-use tokens/IP checks. And those who refused to accept the digital informed consent, the file was closed from that page; another person was selected from that same academic program to ensure adequate representation across programs and academic years. The data were collected from April 2025 to July 2025 using a validated and pre-designed questionnaire.

Research tool: The research team conducted a pilot test of the questionnaire with 33 students to evaluate validity and reliability, and to establish completion time and question clarity before adjusting based on the feedback received. The questionnaire achieved a reliability score of 0.69 through Cronbach’s alpha; the value is slightly below the conventional threshold of 0.70. The instrument’s reliability was strengthened through pilot testing and demonstrated validity *via* significant correlations (<0.05) in the Content Validity Index (CVI) before data collection. The questionnaire included 29 questions, and approximately 6 min are required to complete the survey. Survey tool comprised four sections: The first section gathered basic sociodemographic data through five questions about age, marital status, academic year, skin color, and skin type while the second section assessed sun exposure and protection knowledge with nine questions where each positive answer scored 10 points each negative answer subtracted 5 points and not knowing scored 1 point, Each positive answer scored 10 points, each negative answer subtracted 5 points, and not knowing scored 1 point. These negative marking weightings and smaller scores for “don’t know. This approach ensures respondents are incentivized for correct knowledge and discourages random guessing, improves test validity, and aims to ensure scores reflect correct actual knowledge (Ndu et al., 2016). The thresholds (poor < 50, moderate 50–75, good > 75) were consistent with previous public health KAP frameworks. Total scores below 50 indicate poor knowledge, between 50–75 indicate knowledgeable, and scores above 75 indicate good knowledge. The second research component assessed sun exposure attitudes and protection habits through nine scale-based items ranging from 0 to 2 points, which indicate disagreement to agreement respectively, and results showed negative attitudes for scores under 50 and positive attitudes for scores above 50. The classification of practice performance is aligned with knowledge evaluation, where totals below 50 indicate poor practice, 50–75 reflect adequate practice, and scores above 75 represent good practice after practicing six questions (Wahidiyat et al., 2021).

### Ethical issues

Researchers obtained ethical clearance (ECM#2025-701) from the Research Ethics Committee at King Khalid University. Before proceeding with data collection, the study objectives, the investigators’ contact details, and the purpose of the study were provided on the first screen of the questionnaire. All participants were requested to carefully read and understand the provided study information. After confirming their understanding and willingness to participate, they were asked to provide digital informed consent. Once consent was given, the self-administered questionnaire was opened for completion. Participants who declined to provide consent were automatically exited from the page.

### Statistical analyses

were executed using SPSS version 27 for Windows, and descriptive statistics provided frequency and percentage data for each response. Descriptive statistics (frequencies, percentages) summarized categorical groupings, while inferential tests compared distributions across grouped variables, including academic year *versus* age group and KAP categories; Effect size estimates (Cramer’s V) were calculated to quantify the strength of association between categorical variables. Additionally, Pearson’s correlation coefficients are reported with corresponding 95% confidence intervals to enhance statistical transparency.

## Results

**Figure 1:**
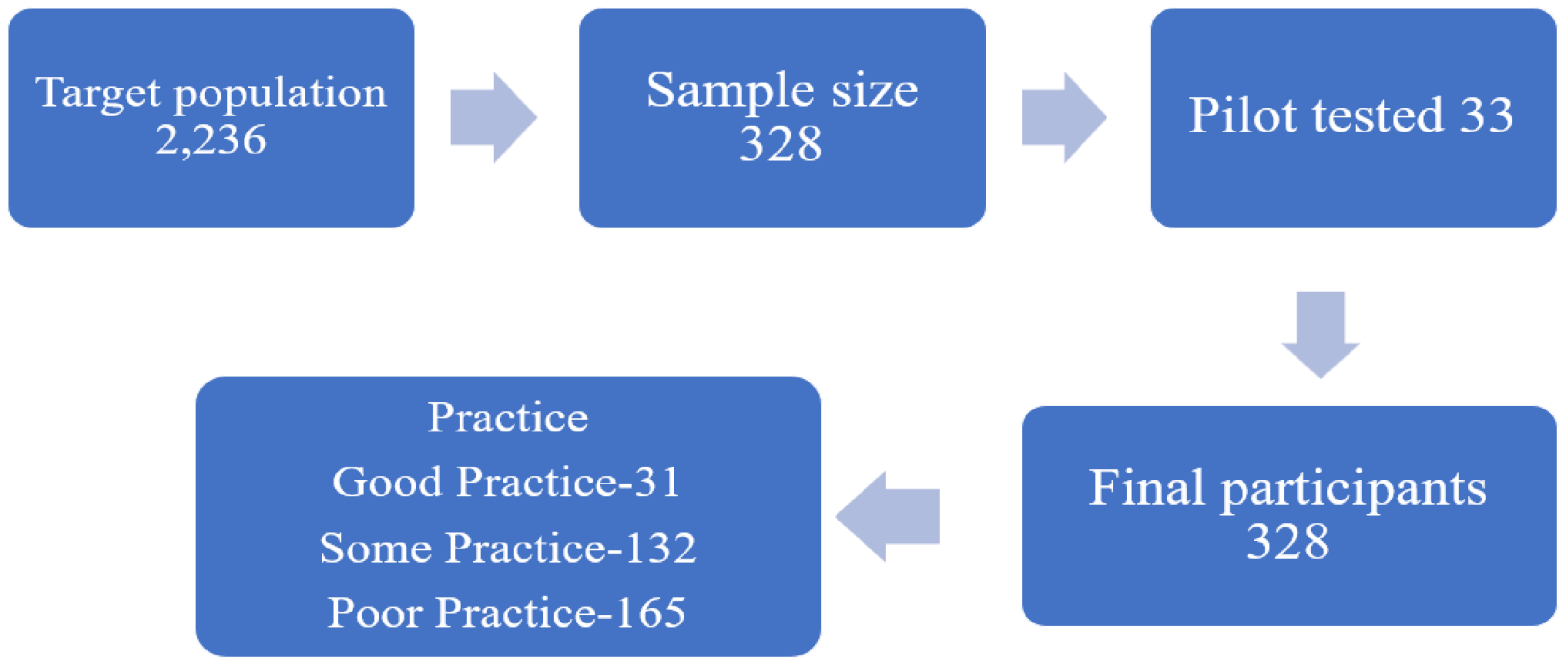
Characteristics and selection stages of the study participants (*N* = 328).

Out of the targeted 2,236 population, the study included 328 participants (Fig. 1), the majority of them were 20–24 years old 165, 50.3%), followed by >25 years (85, 25.9%) and under 19 years 78, 23.8%). Most participants were unmarried (264, 80.5%), of whom 58.0% (153) were aged between 20 and 24 years. A total of 55 participants (16.8%) were married, and (44, 80%) of this group were 25 years old. Only a few respondents were divorced (4, 1.2%) or widowed (5, 1.5%), making them the least represented categories. In terms of academic standing, fourth-year students constituted the largest proportion of the sample, 161 (49.1%), followed by first-year students, 76 (23.2%). A statistically significant association was found between academic year and age group (*p* < 0.001), indicating a meaningful relationship between these variables.

Regarding skin color and type, intermediate skin color was most common (188, 57.3%), followed by fair skin (118, 36.0%) and black skin (22, 6.7%). The mixed skin type predominated 124 (37.8%), while oily (96; 29.3%), normal (75; 22.9%), and dry (33; 10.1%) skin types were also observed. A statistically significant difference was noted among skin type and age categories (*p* < 0.05) (Table 1).

**Table 1:** Sociodemographic characteristics of the study participants (*N* = 328).

Variable		Age group								*P* value
		<19 Years		20–24 Years		≥25 Years		Total		
		No	%	No	%	No	%	No	%	
Social status	Unmarried	76	28.8%	153	58.0%	35	13.3%	264	100%	
	Married	1	1.8%	10	18.2%	44	80.0%	55	100%	
	Divorced/ Separated	0	0.0%	0	0.0%	4	100.0%	4	100%	
	Widow	1	20.0%	2	40.0%	2	40.0%	5	100%	
Academic year	1^st^ year	49	64.5%	14	18.4%	13	17.1%	76	100%	0.0001^*^
	2^nd^ year	20	40.8%	26	53.1%	3	6.1%	49	100%	
	3^rd^ year	7	16.7%	31	73.8%	4	9.5%	42	100%	
	4^th^ year	2	1.2%	94	58.4%	65	40.4%	161	100%	
Skin color	Black	5	22.7%	9	40.9%	8	36.4%	22	100%	0.462^#^
	Fair	27	22.9%	66	55.9%	25	21.2%	118	100%	
	Intermediate	46	24.5%	90	47.9%	52	27.7%	188	100%	
Skin type	Normal	23	30.7%	29	38.7%	23	30.7%	75	100%	
	Dry	8	24.2%	19	57.6%	6	18.2%	33	100%
	Mixed	27	21.8%	73	58.9%	24	19.4%	124	100%	0.05^*^
	Oily	20	20.8%	44	45.8%	32	33.3%	96	100%
Total		78	23.8%	165	50.3%	85	25.9%	328	100%	

**Notes.**

An asterisk (*) means statistically significant (*p* < 0.009), # means not significant.

The study participants demonstrated a strong awareness of sun exposure risks at high altitudes. Among the 328 respondents, 253 (77.1%) recognized that sun rays are more intense at higher elevations, with the 20–24-year age group showing the highest level of awareness, 124 (49.0%), and fourth-year students representing the most informed academic group, 125 (49.4%). Similarly, 239 (72.9%) participants acknowledged that UV radiation is stronger at high altitudes, with 48.1% of this awareness concentrated among those aged 20–24 years. However, 17 (5.2%) participants lacked knowledge about the increased strength of sunlight, and 72 (22.0%) were unaware of elevated UV intensity at high altitudes (Table 2).

**Table 2:** Participants’ knowledge level about sun protection *vs* their academic level.

Knowledge		Academic level										*p*-value
		1st Year		2nd year		3rd year		4th year		Total		
		No	%	No	%	No	%	No	%	No	%	
Stronger sun rays at high altitude	Yes	59	23.3%	39	15.4%	30	11.9%	125	49.4%	253	100%	0.232
	No	16	27.6%	9	15.5%	7	12.1%	26	44.8%	58	100%	
	Don’t know	1	5.9%	1	5.9%	5	29.4%	10	58.8%	17	100%	
High UV intensity at high altitude	Yes	54	22.6%	38	15.9%	28	11.7%	119	49.8%	239	100%	0.172
	No	21	29.2%	10	13.9%	9	12.5%	32	44.4%	72	100%	
	Don’t know	1	5.9%	1	5.9%	5	29.4%	10	58.8%	17	100%	
Usage of Sun protectors	No	25	22.7%	15	13.6%	9	8.2%	61	55.5%	110	100%	0.228
	Yes	51	23.4%	34	15.6%	33	15.1%	100	45.9%	218	100%	
Using sun protectors before 30 causes skin to darken	No	40	27.2%	24	16.3%	18	12.2%	65	44.2%	147	100%	0.31^#^
	Yes	36	19.9%	25	13.8%	24	13.3%	96	53.0%	181	100%	
Intense UV radiation causes skin to age quickly	No	27	30.3%	17	19.1%	9	10.1%	36	40.4%	89	100%	0.08^#^
	Yes	49	20.5%	32	13.4%	33	13.8%	125	52.3%	239	100%	
Stay in the shade to minimize the risk from intense sun rays	No	38	24.7%	24	15.6%	20	13.0%	72	46.8%	154	100%	0.87^#^
	Yes	38	21.8%	25	14.4%	22	12.6%	89	51.1%	174	100%	
Dark clothing protects you from the sun more than light clothing	No	55	21.3%	40	15.5%	32	12.4%	131	50.8%	258	100%	0.41^#^
	Yes	21	30.0%	9	12.9%	10	14.3%	30	42.9%	70	100%	
Good sun protection is required when the intense UV rays	No	25	29.8%	14	16.7%	6	7.1%	39	46.4%	84	100%	0.15^#^
	Yes	51	20.9%	35	14.3%	36	14.8%	122	50.0%	244	100%	
Morning sunbathing for at least 5–30 min per day is good for health	No	21	25.9%	15	18.5%	11	13.6%	34	42.0%	81	100%	0.49^#^
	Yes	55	22.3%	34	13.8%	31	12.6%	127	51.4%	247	100%	
Children must use the sunscreen with <30SPF	No	43	26.7%	25	15.5%	21	13.0%	72	44.7%	161	100%	0.39^#^
	Yes	33	19.8%	24	14.4%	21	12.6%	89	53.3%	167	100%	
Once your skin is tan, there’s no need to use sunscreen	No	46	26.3%	26	14.9%	24	13.7%	79	45.1%	175	100%	0.39^#^
	Yes	30	19.6%	23	15.0%	18	11.8%	82	53.6%	153	100%	
	Total	76	23.2%	49	14.9%	42	12.8%	161	49.1%	328	100%	

**Notes.**

An asterisk (*) means statistically significant (*p* < 0.009), # means not significant.

In terms of protective behavior, 218 (66.5%) participants reported using sun protection products, with the highest usage observed among the 20–24-year-old group, 123 (56.4%). In contrast, non-usage was most frequent among the 25-year-old group (40, 36.4%), contributing to an overall 110 (33.5%) of participants who did not use any sun protection. Despite generally sound knowledge of the hazards associated with high-altitude sunlight, many participants, particularly older students, failed to consistently apply protective measures (Table 2).

Knowledge levels also varied by academic standing. More than half of the respondents, 181 (55.2%), believed that using sun protection before the age of 30 leads to skin darkening, a misconception most prevalent among fourth-year students, 96 (53.0%). A total of 239 participants (72.9%) agreed that intense UV exposure accelerates skin aging, with fourth-year students 125 (52.3%) again showing the highest rate of agreement. Likewise, 174 participants (53.0%) acknowledged that staying in the shade reduces the risks of sun exposure, with 89 fourth-year students (51.1%) supporting this view (Table 2).

The majority of respondents, 258 (78.7%), believed that dark-colored clothing offers better sun protection than light clothing, a view most strongly supported by fourth-year students, 131 (50.8%). Several misconceptions persisted, including the belief that brief morning sun exposure (5–30 min) is beneficial 247 (75.3%) and that sunscreen becomes unnecessary once the skin is tanned 153 (46.6%). Overall, while participants demonstrated a generally good understanding of sun protection, senior students continued to hold certain misconceptions. Most *p*-values for knowledge-related questions were >0.05, indicating no statistically significant differences in knowledge levels across academic years (Table 2).

Participant responses regarding sun exposure habits and protective measures during academic years revealed considerable variability. Enjoyment of sunbathing was reported by 25.6% of participants, with 4th-year students expressing the highest level of agreement at 53.6%; however, 106 individuals (32.3%) disagreed with enjoying sunbathing, and 138 (42.1%) remained neutral. Notably, sunbathing preferences differed significantly across groups (*p* = 0.015) (Table 3).

**Table 3:** Attitude of the participants towards sun exposure and sun protection at high altitudes.

Attitude		Academic year										*p*-value
		1st Year		2nd year		3rd year		4th Year		Total		
		No	%	No	%	No	%	No	%	No	%	
I like sunbathing	Disagree	25	23.6%	27	25.5%	10	9.4%	44	41.5%	106	100%	0.015^*^
	Neutral	30	21.7%	16	11.6%	20	14.5%	72	52.2%	138	100%	
	Agree	21	25.0%	6	7.1%	12	14.3%	45	53.6%	84	100%	
I don’t like being tan	Disagree	33	30.0%	16	14.5%	18	16.4%	43	39.1%	110	100%	0.17^#^
	Neutral	23	21.5%	17	15.9%	12	11.2%	55	51.4%	107	100%	
	Agree	20	18.0%	16	14.4%	12	10.8%	63	56.8%	111	100%	
I don’t feel good when sunbathing for long periods	Disagree	20	25.3%	11	13.9%	9	11.4%	39	49.4%	79	100%	0.99^#^
	Neutral	14	21.9%	9	14.1%	9	14.1%	32	50.0%	64	100%	
	Agree	42	22.7%	29	15.7%	24	13.0%	90	48.6%	185	100%	
What I like about using sunscreen	Disagree	25	33.8%	7	9.5%	4	5.4%	38	51.4%	74	100%	0.094^#^
	Neutral	19	20.2%	15	16.0%	14	14.9%	46	48.9%	94	100%	
	Agree	32	20.0%	27	16.9%	24	15.0%	77	48.1%	160	100%	
Are the future results of sunscreen worth the trouble of using it every two hours	Disagree	17	27.9%	5	8.2%	5	8.2%	34	55.7%	61	100%	0.41^#^
	Neutral	21	22.6%	13	14.0%	15	16.1%	44	47.3%	93	100%	
	Agree	38	21.8%	31	17.8%	22	12.6%	83	47.7%	174	100%	
I worry about sunburn when I sunbathe	Disagree	22	45.8%	4	8.3%	3	6.3%	19	39.6%	48	100%	0.009^*^
	Neutral	12	19.0%	10	15.9%	8	12.7%	33	52.4%	63	100%	
	Agree	42	19.4%	35	16.1%	31	14.3%	109	50.2%	217	100%	
I prefer to be in the shade rather than in the sun in the middle of the day	Disagree	16	47.1%	6	17.6%	4	11.8%	8	23.5%	34	100%	0.009^*^
	Neutral	13	25.0%	6	11.5%	9	17.3%	24	46.2%	52	100%	
	Agree	47	19.4%	37	15.3%	29	12.0%	129	53.3%	242	100%	
	Total	76	23.2%	49	14.9%	42	12.8%	161	49.1%	328	100%	

**Notes.**

An asterisk (*) means statistically significant (*p* < 0.009), # means not significant.

A substantial portion of respondents, 111 (33.8%), indicated negative attitudes toward tanning, as reflected by agreement with the statement ”I don’t like being tan”, but this trend was not statistically significant (*p* > 0.17). Concern about sunburn was pronounced among participants, with 217 (66.2%) expressing worry; this response reached statistical significance (*p* < 0.009). The preference for remaining in shaded areas was prevalent, as 242 respondents (73.8%) agreed with this practice, which was also statistically significant (*p* < 0.009) (Table 3).

Opinions on sunscreen usage were divided: 160 participants (48.8%) agreed or strongly agreed with liking sunscreen use, while 74 (22.6%) expressed dislike. Collectively, these findings demonstrate overall positive attitudes toward sun protection, though significant variation exists in perceived sunburn risks, sunbathing enjoyment, and the preference for shade among academic groups (Table 3)

Participants’ approaches to sun exposure and protective behaviors were evaluated through survey responses. A substantial proportion, 210 (64.0%) individuals strongly disagreed with the use of umbrellas for sun protection, while only 14 (4.3%) participants expressed strong agreement toward this method. Notably, 4th-year students demonstrated the highest level of disagreement, with 101 (48.1%) students indicating strong disapproval (Table 4).

**Table 4:** Participants’ practice towards sun exposure and sun protection.

Practice		Academic year										*p*-value
		1st Year		2nd year		3rd year		4th Year		Total		
		No	%	No	%	No	%	No	%	No	%	
Do you use the umbrella during the day when outside	Strongly disagree	52	24.8%	33	15.7%	24	11.4%	101	48.1%	210	100%	0.38
	Disagree	13	25.0%	9	17.3%	10	19.2%	20	38.5%	52	100%	
	Neutral	7	20.0%	3	8.6%	5	14.3%	20	57.1%	35	100%	
	Agree	1	5.9%	1	5.9%	3	17.6%	12	70.6%	17	100%	
	Strongly agree	3	21.4%	3	21.4%	0	0.0%	8	57.1%	14	100%	
Do you use a head covering during the day	Strongly disagree	18	25.7%	13	18.6%	7	10.0%	32	45.7%	70	100%	0.96
	Disagree	8	23.5%	4	11.8%	7	20.6%	15	44.1%	34	100%	
	Neutral	11	18.0%	9	14.8%	8	13.1%	33	54.1%	61	100%	
	Agree	11	21.6%	8	15.7%	7	13.7%	25	49.0%	51	100%	
	Strongly agree	28	25.0%	15	13.4%	13	11.6%	56	50.0%	112	100%	
Do you avoid the sun in the middle of the day	Strongly disagree	10	38.5%	2	7.7%	4	15.4%	10	38.5%	26	100%	0.15
	Disagree	8	32.0%	3	12.0%	3	12.0%	11	44.0%	25	100%	
	Neutral	20	30.8%	6	9.2%	8	12.3%	31	47.7%	65	100%	
	Agree	25	21.6%	22	19.0%	17	14.7%	52	44.8%	116	100%	
	Strongly agree	13	13.5%	16	16.7%	10	10.4%	57	59.4%	96	100%	
Do you wear long clothes during the day	Strongly disagree	17	34.0%	13	26.0%	3	6.0%	17	34.0%	50	100%	0.006^*^
	Disagree	15	32.6%	6	13.0%	8	17.4%	17	37.0%	46	100%	
	Neutral	10	13.0%	8	10.4%	9	11.7%	50	64.9%	77	100%	
	Agree	18	23.7%	14	18.4%	12	15.8%	32	42.1%	76	100%	
	Strongly agree	16	20.3%	8	10.1%	10	12.7%	45	57.0%	79	100%	
Do you use sunscreen during the day	Strongly disagree	24	29.6%	8	9.9%	9	11.1%	40	49.4%	81	100%	0.08
	Disagree	9	24.3%	8	21.6%	6	16.2%	14	37.8%	37	100%	
	Neutral	10	20.0%	5	10.0%	5	10.0%	30	60.0%	50	100%	
	Agree	23	29.5%	10	12.8%	13	16.7%	32	41.0%	78	100%	
	Strongly agree	10	12.2%	18	22.0%	9	11.0%	45	54.9%	82	100%	
Do you use sunglasses during the day	Strongly disagree	25	33.3%	12	16.0%	12	16.0%	26	34.7%	75	100%	0.10
	Disagree	15	30.6%	6	12.2%	7	14.3%	21	42.9%	49	100%	
	Neutral	10	17.5%	7	12.3%	8	14.0%	32	56.1%	57	100%	
	Agree	12	20.7%	8	13.8%	9	15.5%	29	50.0%	58	100%	
	Strongly agree	14	15.7%	16	18.0%	6	6.7%	53	59.6%	89	100%	
	Total	76	23.2%	49	14.9%	42	12.8%	161	49.1%	328	100%	

**Notes.**

An asterisk (*) means statistically significant (Cramer’s V D 0.331; *p* < 0.001), # means not significant.

Responses regarding the use of head coverings were more evenly distributed, as 112 (34.1%) participants strongly supported their use, contrasted by 70 (21.3%) individuals who strongly opposed the practice. The majority of respondents, 212 (64.9%) individuals, agreed or strongly agreed with avoiding sun exposure during midday hours; this preventive behavior was particularly prominent among 4th-year students, with 57 (59.4%) students displaying the highest agreement. The trend suggests that midday sun avoidance is a commonly adopted strategy for sun protection.

A statistically significant association (*p* < 0.006) was found between wearing long clothes and participants’ responses, with 155 individuals (47.6%) agreeing or strongly agreeing to wearing long clothing during daytime, and 4th-year students 45 (57.0%) reporting the strongest agreement; meanwhile, 50 (15.2%) respondents strongly disagreed with the practice. Sunscreen use exhibited less consistency, as only 160 (48.8%) participants reported regular daytime usage, peaking among 4th-year students at 45 (54.9%) individuals, while 81 (24.7%) participants strongly disagreed with daytime sunscreen application (Table 4).

Sunglasses use revealed a similarly variable pattern, with 147 participants (44.8%) agreeing or strongly agreeing to daytime wear, and the highest agreement among 4th-year students (53 individuals, 59.6%), while 75 participants (22.9%) strongly disagreed. Overall, these findings indicate that behaviors such as midday sun avoidance and wearing long clothing are prevalent among participants, whereas umbrella and sunscreen use remain limited, underscoring the need for enhanced sun protection practices (Table 4).

The mean knowledge score was 45.82 ± 31.97, indicating considerable variability in respondents’ understanding of sun protection concepts. The mean practice score was 51.10 ± 20.08, suggesting that while many participants engaged in some protective behaviors, consistency remained moderate. The mean attitude score was relatively higher at 66.11 ± 19.97, reflecting generally positive perceptions and beliefs toward sun safety.

The relationship between participants’ knowledge levels and their corresponding practice and attitude scores regarding sun exposure and protection. For practice, participants with good knowledge achieved the highest mean practice score (85.12; 95% CI [82.46–87.79]), followed by those with average knowledge (63.65; 95% CI [62.46–64.84]) and poor knowledge (34.66; 95% CI [32.85–36.47]). The association between knowledge and practice was positive but not statistically significant (Cramer’s *V* = 0.272; *p* > 0.219), indicating that higher knowledge levels were not consistently reflected in better protective behaviors (Table 5).

**Table 5:** Participants’ knowledge, attitude, and practice level regarding sun exposure and use of sun protection measures (*N* = 328).

		Knowledge							95% Confidence interval for mean scores		Effect size	*p*-value
		Good knowledge		Average knowledge		Poor knowledge		Mean score				
		No	%	No	%	No	%		Lower	Upper	(Cramer’s V)	
Practice	Good practice	10	13.7%	10	9.0%	11	7.6%	85.12	82.46	87.79		
	Average practicing	33	45.2%	55	49.5%	44	30.6%	63.65	62.463	64.84	0.272	0.219
	Poor practice	30	41.1%	46	41.4%	89	61.8%	34.66	32.85	36.47		
	Total	73	100%	111	100%	144	100%		
	Negative	6	8.2%	12	10.8%	53	36.8%	74.56	73.19	75.93		
Attitude	Positive	67	91.8%	99	89.2%	91	63.2%	35.49	32.19	38.79	0.331	0.001^*^
	Total	73	100%	111	100%	144	100%					

**Notes.**

An asterisk (*) means statistically significant (Cramer’s V D 0.331; *p* < 0.001), # means not significant.

For attitude, participants with good knowledge demonstrated a markedly higher proportion of positive attitudes (91.8%) compared to those with poor knowledge (63.2%). The mean attitude score was significantly higher among those with good knowledge (74.56; 95% CI [73.19–75.93]) than among those with poor knowledge (35.49; 95% CI [32.19–38.79]). This relationship was statistically significant (Cramer’s *V* = 0.331; *p* < 0.001), suggesting that higher knowledge is strongly associated with more favorable attitudes toward sun protection. The results indicate that while improved knowledge significantly enhances attitudes toward sun protection, it does not necessarily translate into consistent protective practices (Table 5).

The given table shows correlation coefficients for the relationship among knowledge practices and attitudes. Research found statistically significant positive correlations between knowledge and practice (*r* = 0.224, *p* < 0.001) as well as between knowledge and attitude (*r* = 0.516, *p* < 0.001) and between practice and attitude (*r* = 0.258, *p* < 0.001). As people develop better knowledge, they experience improved attitudes, together with somewhat improved practices. The lower connection between knowledge and practice indicates that although knowledge impacts behavior, people adopt sun protection measures based on other influences, including convenience, cultural beliefs, and personal preferences (Table 6).

**Table 6:** Correlation between the knowledge, practice, and attitude of the participants towards sun exposure and sun protection at high altitudes.

Correlations				
		Knowledge	Practice	Attitude
Knowledge	Pearson correlation	1	0.224^**^	0.516^**^
	Sig. (2-tailed)		<0.001	<0.001
	Sum of squares and cross-products	334,201	46,927	107,528.3
	Covariance	1,022.0	143.5	3,328.8
	N	328	328	328
Practice	Pearson correlation	0.224^**^	1	0.258^**^
	Sig. (2-tailed)	<0.001		<0.001
	Sum of squares and cross-products	46,927	131,831.7	33,877.5
	Covariance	143.5	403.155	103.601
	N	328	328	328
Attitude	Pearson correlation	0.516^**^	0.258^**^	1
	Sig. (2-tailed)	<0.001	<0.001	
	Sum of squares and cross-products	107,528.4	33,877.5	130,379.3
	Covariance	328.8	103.601	398.713
	N	328	328	328
**Confidence intervals**				
	Pearson correlation	Sig. (2-tailed)	95% Confidence Intervals (2-tailed)^a^	
			Lower	Upper
Knowledge - practice	0.224	0.001	0.118	0.324
Knowledge - Attitude	0.515	0.001	0.431	0.590
Practice - Attitude	0.258	0.001	0.154	0.357

**Notes.**

** Correlation is significant at the 0.01 level (2-tailed).

a Estimation is based on Fisher’s r-to-z transformation with bias adjustment.

## Discussion

This study evaluated university students’ understanding and behaviors regarding sun exposure and protection practices in high-altitude environments. Sunlight exposure can cause a range of harmful outcomes, including tanning, sunburn, premature skin aging, and hyperpigmentation, while also increasing the risk of skin cancer. Appropriate sun protection measures are essential to prevent the cumulative effects of chronic UV radiation exposure. To mitigate these risks, it is vital to enhance knowledge, foster positive attitudes, and promote consistent protective behaviors. Sun protection strategies such as regular sunscreen use and lifestyle modifications have gained importance in recent years due to evolving cosmetic preferences and the influence of social and multimedia trends.

This study demonstrated that participants possessed substantial knowledge of the dangers associated with sun exposure and the importance of protective actions. Among the 328 participants, 77.1% correctly recognized that sunlight intensity increases with altitude, while 72.9% acknowledged the greater strength of UV rays in such regions. Approximately 67.1% of participants demonstrated excellent knowledge of sun protection measures, with the highest awareness observed among students aged 20–24 years. These findings align with scientific evidence indicating that UV radiation levels rise with altitude because the thinner atmosphere absorbs less radiation. Comparable results were reported by Buller et al. (2011), who found that young adults in the United States were aware of UV risks but continued to hold misconceptions about sun protection. Similarly, Rezigue et al. (2025) reported high awareness of UV-related risks among university students in Jordan.

This study noted that almost one quarter (22.2%) of the participants had good knowledge of sun exposure and sun protection, but among those, only 13.7% practiced good, 45.2% practiced average, and the remaining practiced poorly. A similar study by Almuqati, Alamri & Almuqati (2019) also documented a discrepancy between awareness and practicing behavior, noting that Saudi female university students displayed strong knowledge of sun protection yet demonstrated limited adherence to protective practices (Almuqati, Alamri & Almuqati, 2019). Likewise, Bahashwan (2024) and Ahmed (2023) found that although the public had high knowledge scores and positive attitudes, their photoprotection behaviors were insufficient. Conversely, Janjani et al. (2019) observed only moderate understanding of UV risks among participants, accompanied by low engagement in protective practices. In contrast, AlGhamdi, AlAklabi & AlQahtani (2016) and Alsudairy et al. (2019) reported generally low public awareness of sun exposure hazards in Saudi Arabia.

Although overall awareness was high in the present study, several misconceptions persisted. A considerable proportion of participants underestimated the intensity of UV radiation at higher altitudes; 5.2% and 22.0% demonstrated poor understanding of these aspects, respectively. More than half (55.2%) mistakenly believed that using sun protection before the age of 30 could cause skin darkening, and 46.6% thought sunscreen was unnecessary once the skin had tanned. AlGhamdi, AlAklabi & AlQahtani (2016) similarly noted that misconceptions about sun protection often led to inadequate preventive behaviors. In this study, 34.1% of participants perceived sunscreen as harmful, a belief most prevalent among fourth-year students, possibly reflecting cultural or religious effects on attitudes toward sun protection. The study results indicate the need for targeted educational interventions to dispel misinformation and reinforce accurate sun safety knowledge.

Most participants in this study recognized the link between sun exposure and skin cancer risk. Approximately 70% expressed concern about developing wrinkles and dark spots, while 66.2% worried about sunburn. These findings are consistent with Almuqati, Alamri & Almuqati (2019), who reported that most students were highly concerned about skin damage, particularly during peak sunlight hours (10:00 a.m.–2:00 p.m.). AlGhamdi, AlAklabi & AlQahtani (2016) similarly found that while most participants understood sun exposure as a cause of tanning, nearly half associated it with premature skin aging. The majority of respondents in both local and international studies (AlGhamdi, AlAklabi & AlQahtani, 2016) acknowledged the relationship between sun exposure and skin cancer.

Attitudes toward sun exposure varied. One-quarter of participants enjoyed sunbathing, two-thirds expressed concern about sunburn, and the remainder remained neutral. The 32.3% who rejected sunbathing reflected awareness of UV risks but still demonstrated inconsistent protective behaviors. These findings correspond with those of Al Aboud et al. (2021), Watson, Holman & Maguire-Eisen (2016), and Bin Dayel (2025), who observed that many young adults continued to sunbathe despite being aware of the risks (Watson, Holman & Maguire-Eisen, 2016). Similarly, Heckman, Coups & Manne (2008) reported that young adults often prioritize tanning for aesthetic reasons over long-term skin health.

Attitudes toward protective behaviors were also mixed. In this study, 53.0% reported shade-seeking behavior, and 78.7% wore dark clothing patterns consistent with the findings of Geller et al. (2003) among young adults. Additionally, 64.9% of respondents agreed or strongly agreed with avoiding sun exposure during peak hours, and 47.6% endorsed wearing long clothing for protection. Almuqati, Alamri & Almuqati (2019) also found that more than half of the students used shade and nearly half wore long-sleeved clothing. In the present study, over one-fifth of participants reported regular sunscreen use, while more than one-third had never applied sunscreen. AlGhamdi, AlAklabi & AlQahtani (2016) reported similar findings, noting that most participants used long sleeves, sunglasses, and shade-seeking behaviors during outdoor activities, whereas sunscreen use remained uncommon. Only 34.1% of participants in the current study strongly supported head coverings, and 48.8% endorsed sunscreen use, indicating a gap between awareness and behavior. Comparable trends were observed by Al Aboud et al. (2021), who found that most respondents sought shade, but only 27.8% used sunscreen, despite high rates of protective clothing use. These findings emphasize the need for culturally adapted implementation of existing sun safety guidelines.

Sun protection behaviors were inconsistently practiced overall. While 66.5% of participants reported using at least one form of sun protection, 33.5% did not engage in any protective behaviors. This pattern mirrors findings by Buller et al. (2011), who observed that only 34% of young adults consistently applied sunscreen despite understanding UV risks. Similarly, Rezigue et al. (2025) found that only 40% of Jordan university students used sunscreen regularly despite high awareness levels. Misconceptions that sunscreen darkens the skin (55.2%) or is unnecessary for tanned skin (46.6%), coupled with the region’s hot climate and cultural attitudes, likely contribute to low adoption rates. AlGhamdi, AlAklabi & AlQahtani (2016) also noted that cultural and environmental factors significantly influence sun protection behaviors in Saudi Arabia.

In the present study, over two-thirds of participants exhibited strong knowledge regarding sun exposure and protection, and 78.4% expressed positive attitudes toward sun safety. However, nearly half (50.3%) demonstrated poor protective practices. These findings corroborate earlier research by Almuqati, Alamri & Almuqati (2019), which revealed that although female students possessed substantial awareness of sun-related risks, they often failed to translate this knowledge into consistent protective behavior. Similarly, Ahmed (2023) reported high knowledge and positive attitudes among the general public but inadequate adherence to photoprotection. Janjani et al. (2019), in contrast, found moderate knowledge and poor behavioral outcomes. AlGhamdi, AlAklabi & AlQahtani (2016) further observed limited public knowledge and low protective behaviors among Saudi adults (Janjani et al., 2019). Collectively, these findings suggest that while awareness is increasing, significant behavioral gaps remain, emphasizing the importance of culturally sensitive educational initiatives.

## Conclusion

This study examined the understanding and practices of female students at King Khalid University regarding sun exposure and protection in a high-altitude environment. Although most participants were aware of UV-related risks, consistent adoption of protective behaviors, particularly sunscreen use, was lacking. The results revealed significant associations between students’ academic year and their levels of knowledge and attitudes toward sun protection. Misconceptions, such as beliefs that sunscreen causes skin darkening or becomes unnecessary after tanning, were major barriers to effective photoprotection.

The findings underscore the need for culturally appropriate educational programs to correct false beliefs and promote consistent sun safety behaviors. Targeted awareness campaigns focusing on proper sunscreen application, the benefits of UV-protective clothing, and avoidance of peak sunlight exposure could enhance students’ protective practices.

### Recommendations

Universities should implement comprehensive educational programs that raise awareness about ultraviolet (UV) exposure risks at high altitudes and dispel misconceptions regarding sun protection practices. Behavioral strategies should promote consistent sunscreen use, appropriate protective clothing, and avoidance of direct sunlight during peak hours. In addition, campus-level policies can support sun safety by providing shaded outdoor spaces and ensuring that sunscreen is easily accessible to students.

### Limitations

The cross-sectional design precludes causal inference, and the inclusion of only female students limits the generalizability of the findings to the wider population. The reliance on self-reported data introduces the potential for recall and social desirability biases. Future qualitative and Long-term follow-up studies are warranted to evaluate the effectiveness of such interventions in improving sun protection behaviors and reducing UV-related health risks, including skin cancer, in high-altitude populations.

Although the study instrument demonstrated moderate internal consistency (Cronbach’s alpha = 0.69), this level is considered acceptable for exploratory KAP research. The questionnaire underwent pilot testing and expert content validation to ensure adequate construct representation. Effect size measures (Cramer’s V and Pearson’s r) with 95% confidence intervals were also included to enhance the robustness of statistical interpretation.

## Supplemental Information

10.7717/peerj.20576/supp-1 Supplemental Information 1 Data

10.7717/peerj.20576/supp-2 Supplemental Information 2 Questionnaire

10.7717/peerj.20576/supp-3 Supplemental Information 3 STROBE Checklist

## Additional Information and Declarations

### Data Availability

The following information was supplied regarding data availability: The data is available in the Supplementary File.
