# Supplementary material for: Assessment of knowledge, attitudes, and practices regarding sun exposure and sun protection among female students living in high-altitude areas, Abha, Saudi Arabia: a cross-sectional study
Source: PeerJ. 2026 Jan 7;14:e20576. doi: 10.7717/peerj.20576 (PMC12790281; doi:10.7717/peerj.20576)
Supplement: Supplemental Information 2 [file peerj-14-20576-s002.pdf]

Assessment of Knowledge, Attitudes, and Practices Regarding Sun Exposure and Sun Protection Among Female Students Living in High-Altitude Areas, Abha, Saudi Arabia: A Cross-Sectional Study

Dear Students,

This study aims to assess knowledge, attitudes, and practices related to sun exposure and protection among female students in Abha, Saudi Arabia. If you choose to participate, you will complete a questionnaire that takes 10–15 minutes. There are no expected risks and no direct benefits. Your answers will remain confidential, with no names or identifying details recorded. Participation is voluntary—you can withdraw at any time of the study without losing any benefits. There are no costs involved. If you crossed 18 years old and willing to spent 10 minutes please accept the consent to participate.

☐ I agree to participate.

☐ I do not agree to participate.

Part 1: Social and Demographic Characteristics

1. Sex: ☐ Male ☐ Female

2. Age: ☐ 18–21 ☐ 22–25 ☐ More than 25

3. Social status: ☐ Married ☐ Single ☐ Divorced ☐ Widowed

4. Academic year: ☐ 2nd ☐ 3rd ☐ 4th ☐ 5th

5. Skin color: ☐ Very pale ☐ Pale ☐ Intermediate ☐ Dark

6. Skin type: ☐ Oily ☐ Normal ☐ Mixed ☐ Dry

Part 2: Knowledge (9 Questions)

7. Using UV-A tanning cabins before age 30 increases the risk of melanoma? ☐ Yes ☐ No

8. UV radiation causes your skin to age more quickly? ☐ Yes ☐ No

9. There's no risk of radiation effects if we stay in the shade? ☐ Yes ☐ No

10. Using creams is the best way to protect yourself from the sun? ☐ Yes ☐ No

11. You must use increased sun protection when the UV index is > 3? ☐ Yes ☐ No

12. Dark clothing protects you from the sun more than light clothing? ☐ Yes ☐ No

13. You should sunbathe for at least 1 hour per day? ☐ Yes ☐ No

14. Children should use sunscreen with a protection factor ≥ 30? ☐ Yes ☐ No

15. Once your skin is tan, there's no need to use sunscreen? ☐ Yes ☐ No

Part 3: Attitudes (9 Questions)

16. I like sunbathing. ☐ Agree ☐ Indifferent ☐ Disagree

17. I like being tan. ☐ Agree ☐ Indifferent ☐ Disagree

18. I feel good when I'm sunbathing. ☐ Agree ☐ Indifferent ☐ Disagree

19. I don't like using sunscreen. ☐ Agree ☐ Indifferent ☐ Disagree

20. It's worth the effort to use sunscreen. ☐ Agree ☐ Indifferent ☐ Disagree

21. I prefer to be in the shade than in the sun in the middle of the day. ☐ Agree ☐ Indifferent ☐ Disagree

22. I worry about sunburn when I sunbathe. ☐ Agree ☐ Indifferent ☐ Disagree

23. I'm concerned about spots and wrinkles I can get from sunbathing. ☐ Agree ☐ Indifferent ☐ Disagree

24. I worry that I might get skin cancer from sunbathing. ☐ Agree ☐ Indifferent ☐ Disagree

Part 4: Practices (6 Questions)

25. Do you use an umbrella during the day? ☐ Always ☐ Almost always ☐ Sometimes ☐ Almost never ☐ Never

26. Do you use sunglasses during the day? ☐ Always ☐ Almost always ☐ Sometimes ☐ Almost never ☐ Never

27. Do you use a head covering during the day (hijab/shemagh)? ☐ Always ☐ Almost always ☐ Sometimes ☐ Almost never ☐ Never

28. Do you avoid the sun in the middle of the day? ☐ Always ☐ Almost always ☐ Sometimes ☐ Almost never ☐ Never

29. Do you wear long clothes during the day? ☐ Always ☐ Almost always ☐ Sometimes ☐ Almost never ☐ Never

30. Do you use sunscreen during the day? ☐ Always ☐ Almost always ☐ Sometimes ☐ Almost never ☐ Never

تقييم المعرفة والمواقف والممارسات المتعلقة بالتعرض لأشعة الشمس والحماية منها بين الطالبات المقيمات في المناطق المرتفعة، أبها، المملكة العربية السعودية: دراسة مقطعية

عزيزتي الطالبة،

تهدف هذه الدراسة إلى تقييم المعرفة والمواقف والممارسات المتعلقة بالتعرض لأشعة الشمس والحماية منها بين الطالبات في أبها، المملكة العربية السعودية. إذا اخترتِ المشاركة، سيُطلب منكِ تعبئة استبيان يستغرق من 10 إلى 15 دقيقة. لا توجد مخاطر متوقعة ولا فوائد مباشرة من المشاركة. ستبقى إجاباتكِ سرية تمامًا ولن يتم تسجيل أي أسماء أو معلومات تعريفية. المشاركة طوعية بالكامل—يمكنكِ الانسحاب في أي وقت دون أي عقوبة أو فقدان لأي فوائد. لا توجد أي تكاليف مترتبة عليكِ. يجب أن يكون عمركِ 18 عامًا أو أكثر للمشاركة. باختياركِ "أوافق"، فأنتِ تؤكدين أن جميع أسئلتكِ قد تمت الإجابة عليها وأنكِ توافقين بحرية على المشاركة في هذه الدراسة.

☐أوافق على المشاركة.

☐لا أوافق على المشاركة.

الجزء الأول: الخصائص الاجتماعية والديموغرافية

1. الجنس: ☐ ذكر ☐ أنثى
2. العمر: ☐ 18–21 سنة ☐ 22–25 سنة ☐ أكثر من 25 سنة
3. الحالة الاجتماعية: ☐ متزوجة ☐ عزباء ☐ مطلقة ☐ أرملة
4. السنة الدراسية: ☐ الثانية ☐ الثالثة ☐ الرابعة ☐ الخامسة
5. لون البشرة: ☐ شديدة البياض ☐ بيضاء ☐ متوسطة ☐ داكنة
6. نوع البشرة: ☐ دهنية ☐ عادية ☐ مختلطة ☐ جافة

الجزء الثاني: مستوى المعرفة (9 أسئلة)

7. هل استخدام كبائن التسمير بالأشعة فوق البنفسجية قبل سن الثلاثين يزيد من خطر الإصابة بالميلانوما (سرطان الجلد)؟ ☐ نعم ☐ لا
8. هل تسبب الأشعة فوق البنفسجية شيخوخة البشرة المبكرة؟ ☐ نعم ☐ لا
9. هل البقاء في الظل يمنع تمامًا تأثير الأشعة فوق البنفسجية؟ ☐ نعم ☐ لا
10. هل الكريمات الواقية من الشمس هي أفضل وسيلة للحماية من الشمس؟ ☐ نعم ☐ لا
11. هل يجب زيادة الحماية عند التعرض للشمس عندما يكون مؤشر الأشعة فوق البنفسجية أكبر من 3؟ ☐ نعم ☐ لا

12. هل الملابس الداكنة تحمي من الشمس أكثر من الملابس الفاتحة؟ ☐ نعم ☐ لا

13. هل يجب الاستلقاء تحت أشعة الشمس لمدة ساعة على الأقل يوميًا؟ ☐ نعم ☐ لا

14. هل يجب أن يستخدم الأطفال كريم واقٍ من الشمس بعامل حماية 30 فأكثر؟ ☐ نعم ☐ لا

15. هل بمجرد أن تصبح البشرة مسمرة لا تكون هناك حاجة لاستخدام واقي الشمس؟ ☐ نعم ☐ لا

الجزء الثالث: المواقف (9 أسئلة)

16. أحب حمامات الشمس. ☐ أوافق ☐ محايدة ☐ لا أوافق

17. أحب أن تكون بشرتي مسمرة. ☐ أوافق ☐ محايدة ☐ لا أوافق

18. أشعر بالراحة عند التعرض لأشعة الشمس. ☐ أوافق ☐ محايدة ☐ لا أوافق

19. لا أحب استخدام واقي الشمس. ☐ أوافق ☐ محايدة ☐ لا أوافق

20. يستحق الأمر عناء استخدام واقي الشمس. ☐ أوافق ☐ محايدة ☐ لا أوافق

21. أفضل الجلوس في الظل بدلًا من التعرض للشمس منتصف النهار. ☐ أوافق ☐ محايدة ☐ لا أوافق

22. أقلق من الإصابة بحروق الشمس عند حمام الشمس. ☐ أوافق ☐ محايدة ☐ لا أوافق

23. أقلق من ظهور البقع والتجاعيد نتيجة التعرض للشمس. ☐ أوافق ☐ محايدة ☐ لا أوافق

24. أقلق من احتمال إصابتي بسرطان الجلد بسبب حمام الشمس. ☐ أوافق ☐ محايدة ☐ لا أوافق

الجزء الرابع: الممارسات (6 أسئلة)

25. هل تستخدمين المظلة أثناء النهار؟ ☐ دائمًا ☐ غالبًا ☐ أحيانًا ☐ نادرًا ☐ لا أبدًا

26. هل ترتدين النظارات الشمسية أثناء النهار؟ ☐ دائمًا ☐ غالبًا ☐ أحيانًا ☐ نادرًا ☐ لا أبدًا

27. هل تستخدمين غطاء للرأس (حجاب/شماغ) أثناء النهار؟ ☐ دائمًا ☐ غالبًا ☐ أحيانًا ☐ نادرًا ☐ لا أبدًا

28. هل تتجنبين التعرض للشمس منتصف النهار؟ ☐ دائمًا ☐ غالبًا ☐ أحيانًا ☐ نادرًا ☐ لا أبدًا

29. هل ترتدين الملابس الطويلة أثناء النهار؟ ☐ دائمًا ☐ غالبًا ☐ أحيانًا ☐ نادرًا ☐ لا أبدًا

30. هل تستخدمين واقي الشمس أثناء النهار؟ ☐ دائمًا ☐ غالبًا ☐ أحيانًا ☐ نادرًا ☐ لا أبدًا
