# Supplementary material for: Assessment of knowledge, attitudes, and practices regarding sun exposure and sun protection among female students living in high-altitude areas, Abha, Saudi Arabia: a cross-sectional study
Source: PeerJ. 2026 Jan 7;14:e20576. doi: 10.7717/peerj.20576 (PMC12790281; doi:10.7717/peerj.20576)
Supplement: Supplemental Information 3 [file peerj-14-20576-s003.docx]

"**Assessment of Knowledge, Attitudes, and Practices Regarding Sun Exposure and Sun Protection Among Female Students living at high altitude areas, Abha, Saudi Arabia: A Cross-Sectional Study**"

STROBE Statement—checklist of items that should be included in reports of observational studies

|  | Item No | Recommendation | Reported (Yes/No) |
| --- | --- | --- | --- |
| **Title and abstract** | 1 | (*a*) Indicate the study’s design with a commonly used term in the title or the abstract | Yes  Page 1 |
|  |  | (*b*) Provide in the abstract an informative and balanced summary of what was done and what was found | Yes  Page 1 |
| Introduction | | |  |
| Background/rationale | 2 | Explain the scientific background and rationale for the investigation being reported | Yes  Page 2-3 |
| Objectives | 3 | State specific objectives, including any prespecified hypotheses | Yes  Page 4 |
| Methods | | |  |
| Study design | 4 | Present key elements of study design early in the paper | Yes  Page 4 |
| Setting | 5 | Describe the setting, locations, and relevant dates, including periods of recruitment, exposure, follow-up, and data collection | Yes  Page 4 |
| Participants | 6 | (*a*) *Cohort study*—Give the eligibility criteria, and the sources and methods of selection of participants. Describe methods of follow-up  *Case-control study*—Give the eligibility criteria, and the sources and methods of case ascertainment and control selection. Give the rationale for the choice of cases and controls  *Cross-sectional study*—Give the eligibility criteria, and the sources and methods of selection of participants | Descriptive Cross-sectional study  Its outlined  Page 4 |
|  |  | (*b*) *Cohort study*—For matched studies, give matching criteria and number of exposed and unexposed  *Case-control study*—For matched studies, give matching criteria and the number of controls per case | NA |
| Variables | 7 | Clearly define all outcomes, exposures, predictors, potential confounders, and effect modifiers. Give diagnostic criteria, if applicable | Yes  Page 4 &5 |
| Data sources/ measurement | 8* | For each variable of interest, give sources of data and details of methods of assessment (measurement). Describe comparability of assessment methods if there is more than one group | *Yes*  *Page 4 & 5* |
| Bias | 9 | Describe any efforts to address potential sources of bias | Yes  Page 4 |
| Study size | 10 | Explain how the study size was arrived at | Yes  in page 4 |
| Quantitative variables | 11 | Explain how quantitative variables were handled in the analyses. If applicable, describe which groupings were chosen and why | Yes  In page 5 |
| Statistical methods | 12 | (*a*) Describe all statistical methods, including those used to control for confounding | Yes |
|  |  | (*b*) Describe any methods used to examine subgroups and interactions | Yes  In page 5 |
|  |  | (*c*) Explain how missing data were addressed | Yes  Page 4 |
|  |  | (*d*) *Cohort study*—If applicable, explain how loss to follow-up was addressed  *Case-control study*—If applicable, explain how matching of cases and controls was addressed  *Cross-sectional study*—If applicable, describe analytical methods taking account of sampling strategy | Yes  Page 3 |
|  |  | (*e*) Describe any sensitivity analyses | Yes  Page 5 |

Continued on next page

| Results | | | Reported (Yes/No) | |
| --- | --- | --- | --- | --- |
| Participants | 13* | (a) Report numbers of individuals at each stage of study—eg numbers potentially eligible, examined for eligibility, confirmed eligible, included in the study, completing follow-up, and analysed | Yes  Page 6 |  |
|  |  | (b) Give reasons for non-participation at each stage | Yes  Page 6-8 |  |
|  |  | (c) Consider use of a flow diagram | Yes  Page 6 |  |
| Descriptive data | 14* | (a) Give characteristics of study participants (eg demographic, clinical, social) and information on exposures and potential confounders | Yes  Table 1-4 |  |
|  |  | (b) Indicate number of participants with missing data for each variable of interest | No |  |
|  |  | (c) *Cohort study*—Summarise follow-up time (eg, average and total amount) | Na |  |
| Outcome data | 15* | *Cohort study*—Report numbers of outcome events or summary measures over time | *No* |  |
|  |  | *Case-control study—*Report numbers in each exposure category, or summary measures of exposure | *Na* |  |
|  |  | *Cross-sectional study—*Report numbers of outcome events or summary measures | *Yes*  *Page 6-8* |  |
| Main results | 16 | (*a*) Give unadjusted estimates and, if applicable, confounder-adjusted estimates and their precision (eg, 95% confidence interval). Make clear which confounders were adjusted for and why they were included | Yes  Page 13 |  |
|  |  | (*b*) Report category boundaries when continuous variables were categorized | Yes  Page 6-8 |  |
|  |  | (*c*) If relevant, consider translating estimates of relative risk into absolute risk for a meaningful time period | No |  |
| Other analyses | 17 | Report other analyses done—eg analyses of subgroups and interactions, and sensitivity analyses |  |  |
| Discussion | | |  | |
| Key results | 18 | Summarise key results with reference to study objectives | Yes  Page 15-17 |  |
| Limitations | 19 | Discuss limitations of the study, taking into account sources of potential bias or imprecision. Discuss both direction and magnitude of any potential bias | Yes  Page 19 |  |
| Interpretation | 20 | Give a cautious overall interpretation of results considering objectives, limitations, multiplicity of analyses, results from similar studies, and other relevant evidence | Yes  Page 19 |  |
| Generalisability | 21 | Discuss the generalisability (external validity) of the study results | Yes  Page 19 |  |
| Other information | | |  | |
| Funding | 22 | Give the source of funding and the role of the funders for the present study and, if applicable, for the original study on which the present article is based | Yes  Page 20 |  |

*Give information separately for cases and controls in case-control studies and, if applicable, for exposed and unexposed groups in cohort and cross-sectional studies.
